# Postimplantation pocket hematoma increases risk of cardiac implantable electronic device infection: A meta‐analysis

**DOI:** 10.1002/joa3.12516

**Published:** 2021-03-13

**Authors:** Jakrin Kewcharoen, Chanavuth Kanitsoraphan, Sittinun Thangjui, Thiratest Leesutipornchai, Sakditad Saowapa, Apichai Pokawattana, Leenhapong Navaravong

**Affiliations:** ^1^ University of Hawaii Internal Medicine Residency Program Honolulu HI USA; ^2^ Faculty of Medicine Chulalongkorn University Hospital Bangkok Thailand; ^3^ Faculty of Medicine Ramathibodi Hospital Mahidol University Bangkok Thailand; ^4^ Division of Cardiology Rajavithi Hospital Bangkok Thailand; ^5^ Division of Cardiovascular Medicine University of Utah School of Medicine Salt Lake City UT USA

**Keywords:** cardiac implantable electronic device, cardiac implantable electronic device infection, hematoma

## Abstract

**Introduction:**

Several studies have shown an inconsistent relationship between postimplantation pocket hematoma and cardiac implantable electronic device (CIED) infection. In this study, we performed a systematic review and meta‐analysis to explore the effect of postimplantation hematoma and the risk of CIED infection.

**Methods:**

We searched the databases of MEDLINE and EMBASE from inception to March 2020. Included studies were cohort studies, case‐control studies, cross‐sectional studies, and randomized controlled trials that reported incidence of postimplantation pocket hematoma and CIED infection during the follow‐up period. CIED infection was defined as either a device‐related local or systemic infection. Data from each study were combined using the random effects, generic inverse variance method of Der Simonian and Laird to calculate odds ratios (OR) and 95% confidence intervals (CI).

**Results:**

Fourteen studies were included in final analysis, involving a total of 28 319 participants. In random‐effect model, we found that postimplantation pocket hematoma significantly increases the risk of overall CIED infection (OR = 6.30, 95% CI: 3.87‐10.24, *I* ^2^ = 49.3%). There was no publication bias observed in the funnel plot as well as no small‐study effect observed in Egger’s test.

**Conclusions:**

Our meta‐analysis demonstrated that postimplantation pocket hematoma significantly increases the risk of CIED infection. Precaution should be taken during device implantation to reduce postimplantation hematoma and subsequent CIED infection.

## Graphical abstract

Our meta‐analysis included 14 studies, involving 28 319 participants. We demonstrated that postimplantation pocket hematoma significantly increases overall risk of infection (OR = 6.30, 95% CI: 3.87‐10.24, *I* ^2^ = 49.3%).

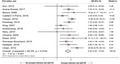

## 1 INTRODUCTION

Approximately 1.5 million people receive cardiovascular implantable electronic devices (CIEDs) worldwide per year.^1^ The use of CIEDs by cardiologists has been increasing because of the expansion of the eligibility criteria for CIEDs in recent guidelines of cardiac arrhythmias.^2^, ^3^ Even though CIED implantation has been shown to improve outcomes in the selected population, the procedure carries risk of complications, such as CIED infection, which is associated with increased morbidity, mortality, hospital length of stay, and substantial financial burden.^4^, ^5^, ^6^ The clinical manifestations of CIED infection can vary from local pocket infection to systemic infection such as endocarditis, bacteremia, or lead infection. The overall incidence of CIED infection ranges from 0.68% to 5.7%,^7^ with increased rate in patients who have comorbidities including diabetes mellitus, end‐stage renal disease, chronic obstructive pulmonary disease, corticosteroid use, malignancy, and heart failure.^8^

Postimplantation pocket hematoma is another common complication after CIED implantation, reported occurring around 1.04% to 16.67%.^9^, ^10^, ^11^, ^12^, ^13^, ^14^, ^15^, ^16^, ^17^, ^18^, ^19^, ^20^, ^21^, ^22^ However, the results from previous studies exploring the effect of postimplantation hematoma on the risk of CIED infection have been inconsistent. Several studies showed that postimplantation hematoma increased the risk of CIED infection after device implantation,^10^, ^12^, ^13^, ^15^, ^16^, ^17^, ^19^, ^20^, ^22^ while others failed to demonstrate such a relationship.^9^, ^11^, ^14^, ^18^, ^21^ Thus, the primary objective of this study was to evaluate the association between postimplantation hematoma and the risk of CIED infection following cardiac implantable electronic device implantation via the systematic review and meta‐analysis.

## 2 METHODS

### 2.1 Search strategy

Two investigators (CK and ST) independently searched for published studies indexed in the MEDLINE and EMBASE databases from inception to March 2020 using a search strategy including the terms “hematoma,” “cardiac implantable electronic device,” and “infection” as described in Data S1. Only full articles in English were included. A manual search for additional pertinent studies using references from retrieved articles was also completed.

### 2.2 Inclusion criteria

The eligibility criteria included the following:

1. Cohort studies (prospective or retrospective), case‐control studies, cross‐sectional studies, and randomized control trials (RCT) reporting incidence of CIED infection following the implantation, comparing between patients with postimplantation hematoma and without postimplantation hematoma
2. Relative risk (RR), odds ratio (OR), hazard ratio (HR) with 95% confidence interval (CI), or sufficient raw data to perform the above calculations were provided. Patients without documented postimplantation hematoma were used as controls

Study eligibility was independently determined by two investigators (CK and ST) and differences were resolved by mutual consensus. The Newcastle‐Ottawa quality assessment scale (NOS) was used. The Newcastle‐Ottawa Scale uses a star system (0 to 9) to evaluate included studies on three domains: recruitment and selection of the participants, similarity and comparability between the groups, and ascertainment of the outcome of interest among cohort and case‐control studies.^23^ Higher scores represent higher quality studies.

### 2.3 Data extraction

A standardized data collection form was used to obtain the following information from each study: title of study, name of the first author, year of publication, country of origin, prevalence and diagnostic method of CIED infection, device type, time of outcome measurement, definition of postimplantation hematoma, and pathogen.

Two investigators (CK and ST) independently performed this data extraction process to ensure accurate data extraction. Any data discrepancy was resolved by reviewing the primary data from the original articles.

### 2.4 Definition

Cardiac implantable electronic device infection was defined as either local/wound infection or systemic infection (bacteremia or infective endocarditis or lead infection/vegetation) or both.^24^

### 2.5 Statistical analysis

We performed a meta‐analysis of the included studies using a random‐effect model. Studies were excluded if they did not report an outcome in each group or did not have enough information available to calculate the OR or RR. We pooled the point estimates of HR, RR, and OR from each study, separately for each type of parameters, using the generic inverse‐variance method of Der Simonian and Laird.^25^ The heterogeneity of effect size estimated across these studies was quantified using the *I* ^2^ statistic. The *I* ^2^ statistic ranges in value from 0% to 100% (*I* ^2^ **< **25%, low heterogeneity; *I* ^2^ **= **25%–50%, moderate heterogeneity; and *I* ^2^ **≥ **50%, substantial heterogeneity).^26^ A sensitivity analysis was performed to assess the influence of the individual studies on the overall results. Publication bias was assessed using a funnel plot and the Egger’s regression test,^27^ with a *P* < .05 being considered significant. All data analyses were performed using STATA SE version 14.2.

### 2.6 Sensitivity analysis

We used a sequential exclusion strategy, as described by Patsopoulos et al, to examine whether overall estimates were influenced by the substantial heterogeneity observed.^28^ We sequentially and cumulatively excluded studies that accounted for the largest share of heterogeneity until I^2^ was less than 50%. We then examined whether OR estimates were consistent.

## 3 RESULTS

### 3.1 Search results

**FIGURE 1:**
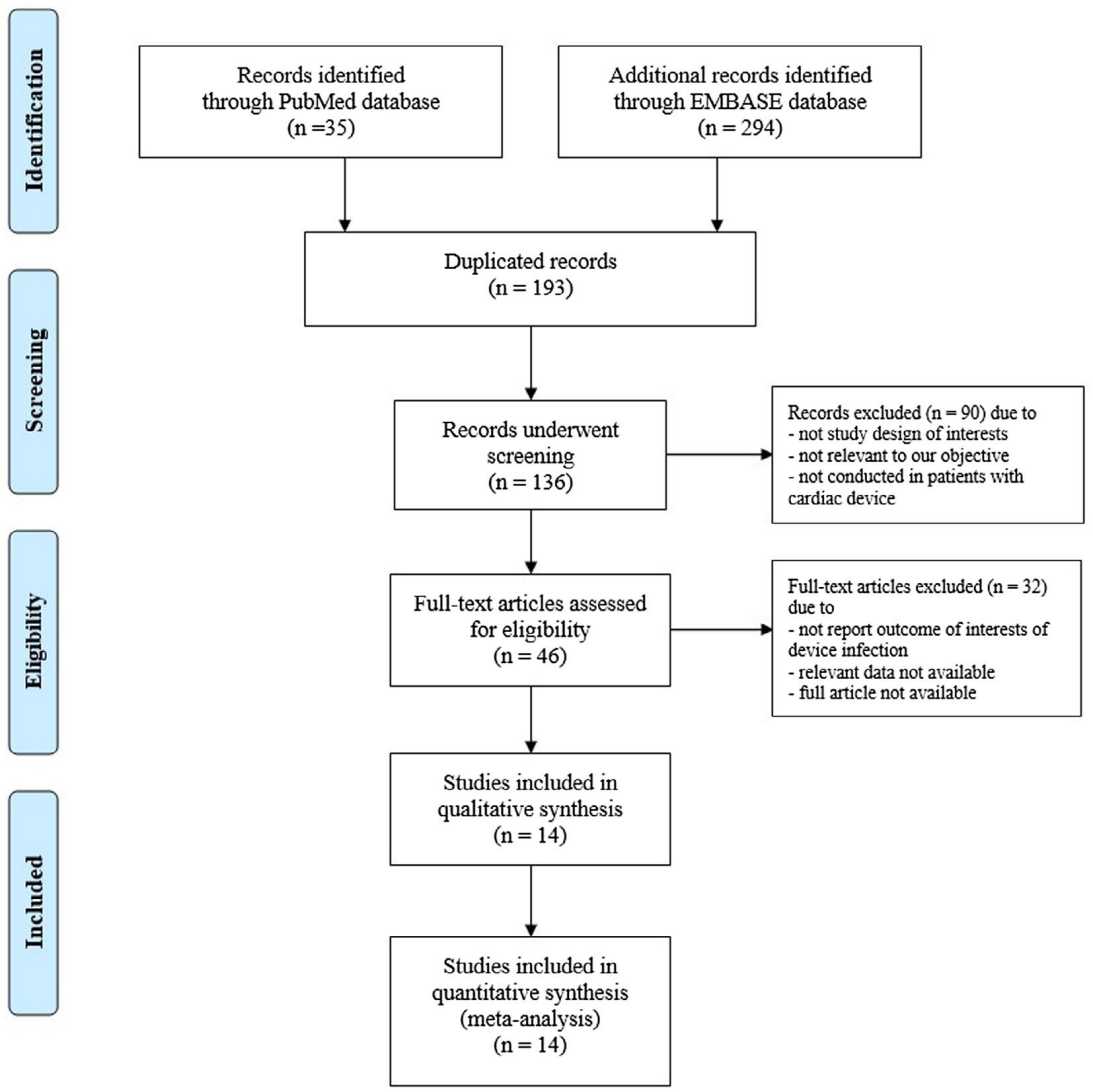
PRISMA flow diagram

Our search strategy yielded 329 potentially relevant articles (294 articles from EMBASE and 35 articles from MEDLINE). After the exclusion of 193 duplicated articles, 136 articles underwent title and abstract review. At this stage, 90 articles were excluded as they were not conducted in patients with cardiac device, not study design of interests, or not relevant to our objective. This left 46 articles for full‐length review. Further 32 studies were excluded as they did not report outcome of interests of device infection, relevant data were not available, or full article was not available. No additional studies were added through the manual search. Therefore, a total of 14 studies were included in the meta‐analysis.^9^, ^10^, ^11^, ^12^, ^13^, ^14^, ^15^, ^16^, ^17^, ^18^, ^19^, ^20^, ^21^, ^22^ The PRISMA flow diagram is demonstrated in Figure 1.

### 3.2 Description of included studies

A total of 14 studies from 2006 to 2018 were included in our meta‐analysis with a total population of 28 319 patients, with 14 898 patients being analyzed as 13 421 patients were excluded from matched case‐control studies. The incidence of CIED infection ranged from 0.68% to 4.56%. The most common pathogen is *Staphylococcus aureus* (31.4%) and coagulate‐negative Staphylococcus (25.3%). A summary of study characteristics is shown in Table 1.

**TABLE 1:** Characteristics of the included studies

First author, year	Study design	Country	Analyzed population (n)	Male (%)	mean age ± SD	Follow‐up time (mo)	PH definition	PH Incidence	Device type	Mean LVEF (%)		DM (%)		CHF (%)		Renal disease (%)		CIED infection incidence (%)	Pathogen (%)
										Infection	No infection	Infection	No infection	Infection	No infection	Infection	No infection		
Ann, 2015	Single‐center, retrospective case‐control study	Korea	36	49.6	61.5 ± 14.2	Mean 6.96 device‐year	N/A	5.55%	PPM (86.5%), ICD (11.2%), CRT (2.3%)	64.8 ± 14.2	61.5 ± 14.2	8.3%	29.2%	8.3%	12.5%	0%	16.6%	0.9%	SA 8.3% CNS16.63% Enterococcus spp. 16.6% E. coli 8.3%
Arana‐Rueda, 2017	Single‐center, prospective cohort study	Spain	570	80	59 ± 14	Median 36 (IQR 18,61)	Any blood collection in the pocket with swelling, pain, or functional impairment	4.56%	ICD	<30 (42.4%)	<30 (59.7%)	42.3%	33.4%	N/A	N/A	3.8%	0.36%	4.6%	CNS 65.3% SA 7.7% GNB 6% Others 9%
Bloom, 2006	Single‐center, retrospective case‐control study	USA	152	77	66.7 ± 12	N/A	N/A	9.86%	PPM (44.5%), ICD (55.5%)	N/A	N/A	42.1%	18.4%	60.5%	39.5%	42.0%	13.0%	1.5%	N/A
Caldero´n‐Parra, 2018	Single‐center, retrospective case‐control study	Spain	132	70.5	median 63 (IQR, 54, 75.5)	At least 12	N/A	16.66%	PPM, ICD, CRT	N/A	N/A	30.3%	34.3%	66.6%	44.4%	27.3%	21.2%	1.4%	CNS 55% SA 21%
Cengiz, 2010	Single‐center, retrospective case‐control study	Turkey	890	57.4	Infected: median 65, control: median 58	Mean 34.8	A palpable mass that protruded ≥ 2 cm anterior to the generator	2.02%	PPM, ICD	N/A	N/A	29.8%	21.0%	N/A	N/A	N/A	N/A	6.4%	CNS 14.0% SA 12.3%
Essebag, 2015	Multi‐center, prospective cohort study	Canada	659	72.7	71.7 ± 10.4	12	PH needing surgical evacuation, resulting in prolonged hospitalization or interruption of anticoagulants	10.01%	CIED	N/A	N/A	18.7%	39.2%	N/A	N/A	N/A	N/A	2.4%	SA 31.3% CNS 25% Other Staphylococcus spp. 6.3%
Klug, 2007	Multi‐center, prospective cohort study	France	6319	59.7	73.4 ± 13.9	12	N/A	5.34%	PPM (92.8%), ICD (7.1%)	N/A	N/A	10.1% (overall population)		N/A	N/A	N/A	N/A	0.7%	CNS 57.1% SA 13.0%
Korkerdsup, 2018	Single‐center, retrospective case‐control study	Thailand	162	67	median 67.5 (IQR, 53 ,75)	N/A	N/A	4.32%	PPM, ICD, CRT	55%	41%	29.6%	24.1%	9.3%	12.0%	7.4%	9.3%	0.9%	CNS 22.2% SA 18.5% P. aeruginosa 7.4%
Nery, 2010	Single‐center, retrospective case‐control study	Canada	96	67.25	68.5 ± 14	N/A	N/A	1.04%	PPM, ICD, CRT	N/A	N/A	33.3%	36.1%	N/A	N/A	N/A	N/A	1.0%	SA 20.8% CNS 12.5% Viridians Streptococci 4.2%
Oliveira, 2009	Single‐center, randomized controlled trial	Brazil	649	46.7	64 ± 15	6	Any swelling of the pocket site	2.53%	PPM, ICD, CRT	50.2 ± 11.4	57.3 ± 26.6	44.4%	18.0%	N/A	N/A	N/A	N/A	2.0%	SA 61.5% CNS 38.5%
Raad, 2012	Single‐center, retrospective case‐control study	USA	72	72	70 ± 10.4	N/A	Palpable swelling of the pocket exceeding the size of the generator	6.94%	PPM, ICD	N/A	N/A	50.0%	33.3%	50.0%	38.9%	22.2%	22.2%	N/A	CNS 33.3% S.aureus 11.1%
Romeyer‐Bouchard, 2009	Single‐center, prospective cohort study	France	303	81.5	70 ± 10	Mean 31 ± 19	N/A	9.57%	CRT	25.8 ± 5	26.3 ± 6	30.7%	22.1%	N/A	N/A	23.1%	1.72%	4.3%	SA 53.8% CNS 15.4% GNB 7.7%
Sadeghi, 2018	Single‐center, retrospective cohort study	Iran	3205	62.3	62.5 ± 16	Mean 27 ± 11	PH needing surgical evacuation, resulting in prolonged hospitalization or interruption of anticoagulants	1.93%	PPM, ICD, CRT	30.0 ± 14	29.0 ± 14	31.8%	20.0%	N/A	N/A	9.4%	0.9%	2.7%	SA and CNS 76.8% Streptococcus spp. and GNB 19.2%
Uslan, 2012	Multi‐center, prospective cohort study	USA	1744	67.8	70.2 ± 13.7	6	N/A	1.26%	PPM, ICD, CRT	37.7 ± 16.5	36.3 ± 16.5	50.0%	29.1%	86.4%	66.8%	18.2%	16.4%	1.3%	SA 14.3% CNS 14.3%

Abbreviations: CIED, cardiac implantable electronic device; CNS, Coagulase‐negative staphylococci; CRT, cardiac resynchronization therapy; GNB, Gram‐negative bacilli; ICD, implantable cardiac device; PH, postimplantation pocket hematoma; PPM, permanent pacemaker; SA, *Staphylococcus aureus*.

### 3.3 Quality assessment of included studies

The NOS of included studies are described in Data S2.

### 3.4 Meta‐analysis results

#### 3.4.1 Postimplantation pocket hematoma and cardiac implantable electronic device infection

**FIGURE 2:**
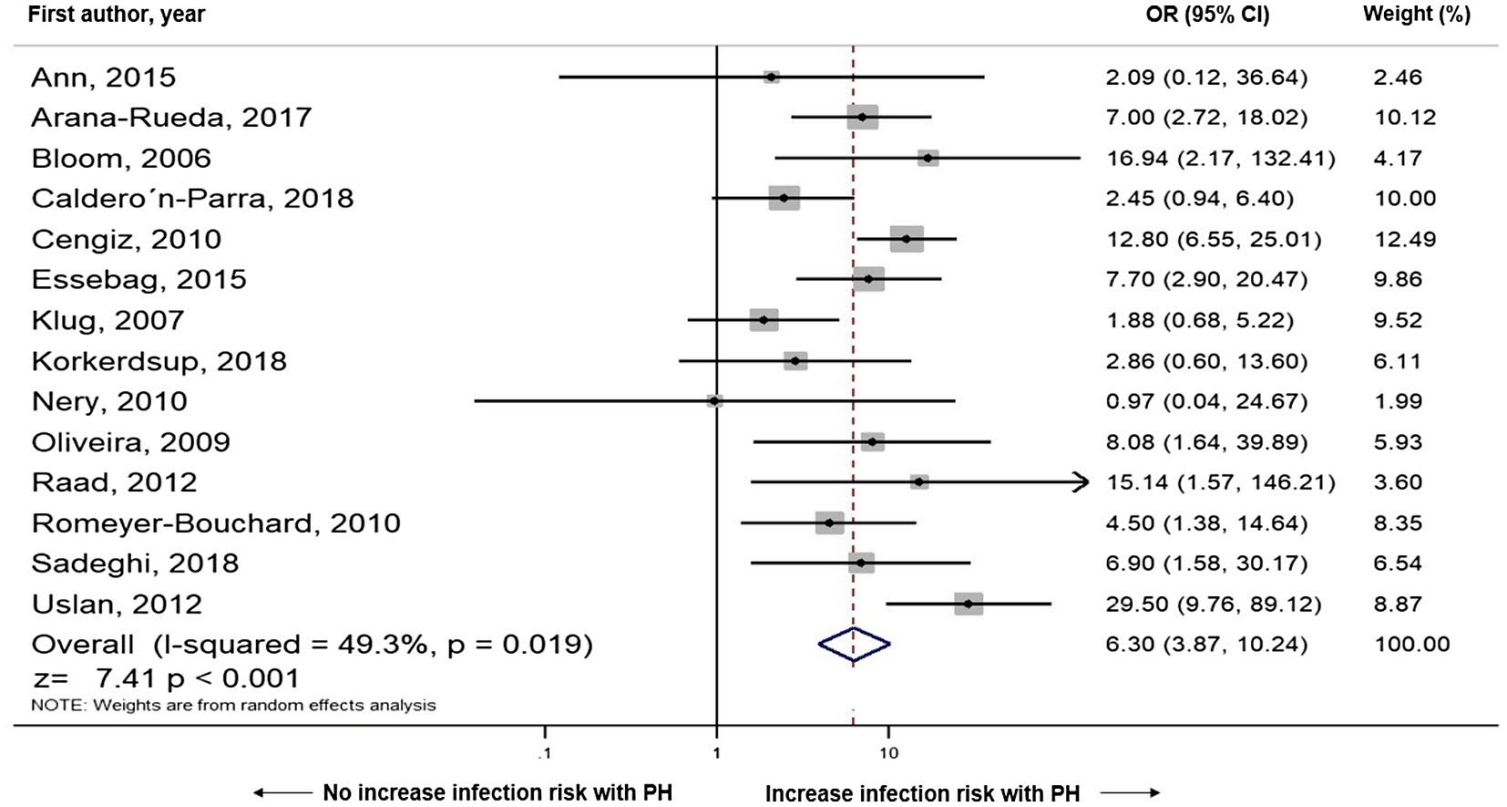
Forest plot demonstrating the association of postoperative hematoma and cardiac implantable electronic device infection

Outcomes regarding the association between postimplantation pocket hematoma and CIED infection were available in all 14 studies. Outcomes extracted from were OR, or raw data to calculate OR, in all 14 studies. There was a significant association between postimplantation pocket hematoma and increased risk of CIED infection (OR = 6.30, 95% CI: 3.87‐10.24, *I* ^2^ = 49.3, *P* < .001). The Forest plot demonstrating the association between postimplantation hematoma and CIED infection is shown in Figure 2.

**FIGURE 3:**
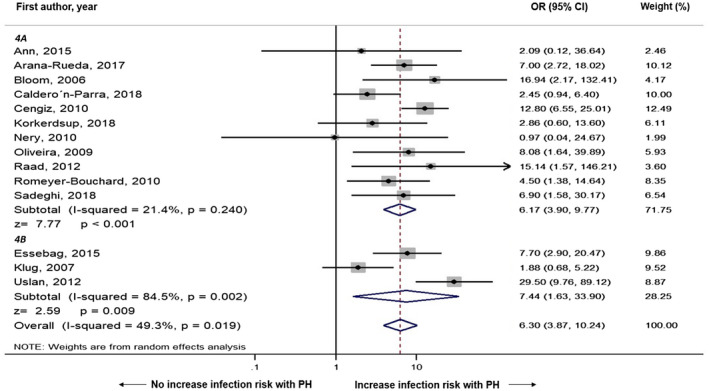
Forest plot demonstrating the association of postoperative hematoma and cardiac implantable electronic device infection with subgroup analysis by number of centers; 4A: Single‐center studies, 4B: Multi‐center studies

**FIGURE 4:**
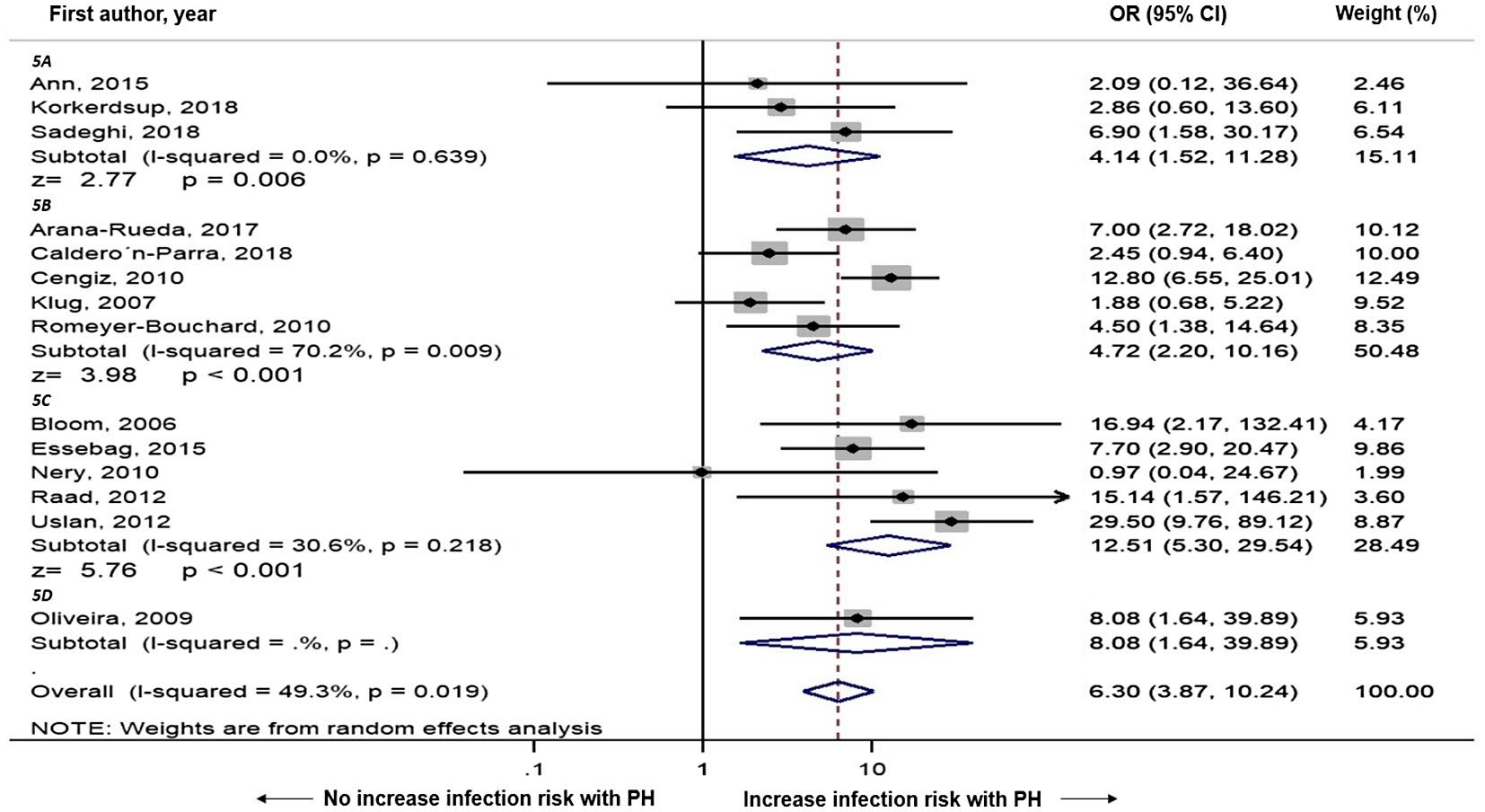
Forest plot demonstrating the association of postoperative hematoma and cardiac implantable electronic device infection with subgroup analysis by region of country of study; 5A: Asia, 5B: Europe, 5C: North America, 5D: South Africa

We performed subgroup analysis based on number of center (single vs multiple), and region of study (Asia vs Europe vs North America vs Africa), which are demonstrated in Figures 3 and 4, respectively. In subgroup analysis by number of centers, multi‐center studies (OR = 7.44, 95% CI: 1.63‐33.90, *I* ^2^ = 84.5%, *P* = .009) and single‐center studies (OR = 6.17, 95% CI: 3.90‐9.77, *I* ^2^ = 21.4%, *P* < .001) both demonstrated association between postimplantation pocket hematoma and risk of CIED infection. In subgroup analysis by country or region, North America (OR  =  12.51, 95% CI: 5.30‐29.54, *I* ^2^ = 30.6%, *P* < .001) demonstrated the strongest association between postimplantation pocket hematoma and risk of CIED infection comparing to Asia (OR = 4.14, 95% CI: 1.52‐11.28, *I* ^2^ = 0.0%, *P* = .006), Europe (OR = 4.72, 95% CI: 2.20‐10.16, *I* ^2^ = 70.2%, *P* < .001), and South Africa (OR = 8.08, 95% CI: 1.64‐39.89, *P* < .001).

#### 3.4.2 Sensitivity analysis

To assess the stability of the results of the meta‐analysis, we conducted a sensitivity analysis for each outcome by excluding one study at a time. For every outcome, none of the results were significantly altered, as the results after removing one study at a time were similar to that of the main meta‐analysis, indicating that our results were robust.

#### 3.4.3 Publication bias

**FIGURE 5:**
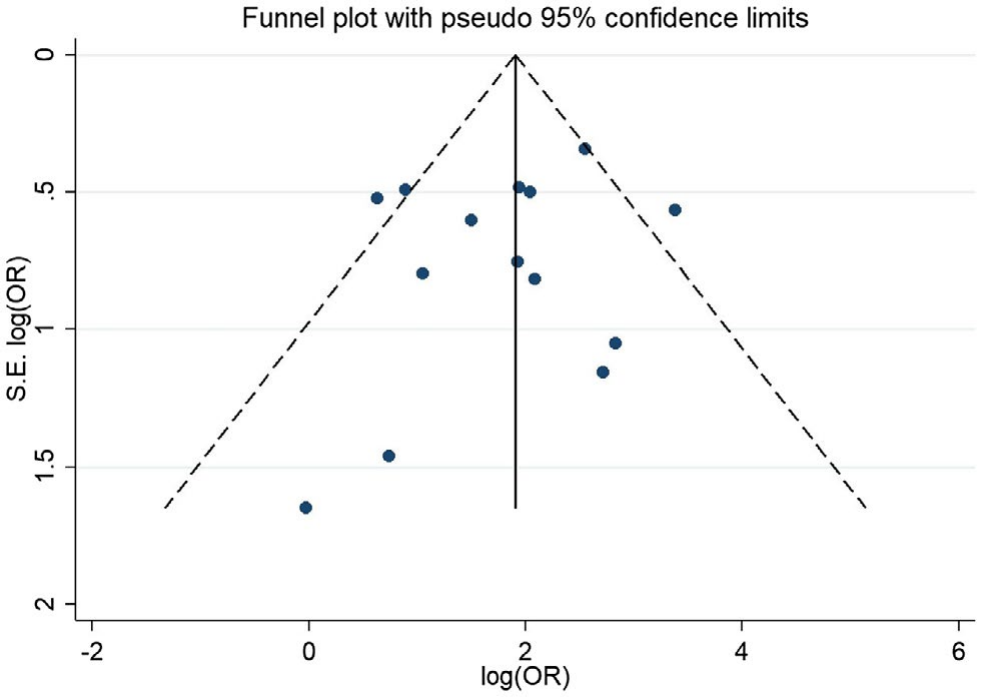
Funnel plot evaluating publication bias of the meta‐analysis

To investigate the effect of potential publication bias on the main outcome, we examined a funnel plot generated from the included studies (Figure 5). The vertical axis represents study size (standard error) while the horizontal axis represents effect size (log odds ratio). From this plot, no bias was observed, as the distribution of studies is symmetrical on both sides of the mean. Egger's test was not significant.^29^, ^30^

## 4 DISCUSSION

The main result from our meta‐analysis showed that postimplantation pocket hematoma is associated with an increased risk of CIED infection up to 6.3‐folds.

Recent systematic review suggested that there is an association between pocket hematoma and risk of wound infection among patients with a CIED.^31^ However, this study mainly focused on pocket infection rather than systemic infection. Additionally, meta‐analysis was not performed and only OR from included studies were reported. This is the first study to explore the association between postimplantation hematoma and the risk of CIED infection following CIED implantation via the systematic review and meta‐analysis.

There has been conflicting data regarding the association between pocket hematoma and the risk of CIED infection. Among the included studies in our systematic review, nine from 14 studies revealed a significant association between postimplantation pocket hematoma and an increased risk of CIED infection.^10^, ^12^, ^13^, ^15^, ^16^, ^17^, ^19^, ^20^, ^22^ Four studies suggested positive correlation, but the results did not reach statistical significance.^9^, ^11^, ^14^, ^21^ Only one study did not demonstrate positive correlation, although the study had a small number of CIED infection events.^18^ In subgroup analysis, there is a slightly stronger association between postimplantation hematoma and risk of CIED infection in multi‐center studies when compared to single‐center studies. In subgroup analysis by region/country of origin, North America demonstrated the strongest association between postimplantation hematoma and risk of CIED infection comparing to Asia, Europe, and South Africa.

The stronger effect size observed in North America region is mainly driven by studies from Bloom et al, Essebag et al, Raad et al, and Uslan et al.^13^, ^15^, ^16^, ^22^ Essebag et al reported relatively high incidence of postimplantation pocket hematoma and CIED infection. The authors defined postimplantation pocket hematoma as hematoma needing surgical evacuation, resulting in prolonged hospitalization or interruption of anticoagulants. By this definition, participants with only significant pocket hematoma would be considered and would be at a higher risk of CIED infection. Compared to studies from other regions, participants in studies by Bloom et al, Raad et al and Uslan et al had more comorbidities such as diabetes mellitus, heart failure, and renal dysfunction (Table 1). These factors are known to be associated with infection. This may increase the risk of CIED infection in patients with pocket hematoma, therefore driving the association strength. In other regions, the lower effect sizes were driven by studies from Ann et al and Korkerdsup et al for Asia region, and Calderón‐Parra et al and Klug et al for Europe region.^9^, ^11^, ^14^, ^21^ In the opposite fashion, there were less participants with significant comorbidities in studies by Ann et al, Korkerdsup et al and Klug et al. Population in study by Calderón‐Parra et al had comparable comorbidities to studies from North America region but were younger. These factors likely contributed to the weaker association of postimplantation pocket hematoma and CIED infection found in other regions.

Patients with cardiovascular disease undergoing CIED implantation commonly have risk factors associated with delayed wound healing and wound infection. For example, advanced age, diabetes mellitus, peripheral artery disease, tobacco use are among the established risk factors for wound complications that are common in patients with cardiovascular conditions.^32^, ^33^, ^34^, ^35^ For CIED implantation specifically, diabetes mellitus, heart failure, and renal failure were shown to strongly increase the risk of device infection. Other factors such as chronic obstructive pulmonary disease, use of immunosuppression and corticosteroid usage were also reported to be associated with CIED infection as well. Additionally, a significant portion of patients undergoing CIED implantation are on antithrombotic medications prior to the procedure, which also substantially increase the risk of bleeding and pocket hematoma.^36^, ^37^, ^38^

The mechanism of postimplantation hematoma leading to CIED infection has been proposed. Although up to 60 to 80% of CIED infections were caused by staphylococcal species, virtually any pathogen can cause the infection, including normal flora.^39^ Hematoma can separate the incision, making the wound vulnerable to bacterial migration through the superficial tissue and into a subsequent deeper layer.^40^ Conversely, compromised wound closure could also lead to hematoma development as well.^41^ Moreover hematoma itself can act as a culture medium for bacterial growth.^42^ There is evidence describing risk factors for pocket hematoma following CIED implantation including heart failure, renal failure, coagulopathy.^36^, ^37^ As these are similar risk factors for CIED infection, it is possible that these populations simply just possess risk profiles for both hematoma and CIED infection rather than having a causal association.

Treatment or prevention of hematoma is not yet standardized. Careful implant technique could reduce the risk of pocket hematoma development. Although anticoagulants are known to increase risk of bleeding and pocket hematoma following the procedure, there is still no clear evidence regarding the management in patients undergoing CIED implantation, and most recommendations are based on expert consensus. Individual factors for bleeding and thrombosis must be taken into consideration when deciding to continue or withhold antithrombotic agents. This leads to great variation in clinical practices.^43^ Previous studies proposed that heparinization, dual antiplatelet therapy, clopidogrel and lack of experience of the implanting physician are associated with the development of pocket bleeding.^37^, ^44^ The Bridge or Continue Coumadin for Device Surgery Randomized Controlled (BRUISE CONTROL) revealed that continuation of warfarin peri‐procedurally was associated with lower bleeding risk compared to bridging to heparin, without significant difference in incidence of thromboembolic events.^45^ European Heart Rhythm Association (EHRA) international consensus document recommends continuing oral anticoagulation in high thromboembolic risk patients (prior embolic event or mechanical valve) and consider stopping prior to surgery in patients with low‐to‐moderate thromboembolic risk (CHA2DS2‐VASc score <4).^46^ Current guideline recommends against heparin “bridging,” including therapeutic low‐molecular‐weight heparin.^47^ Regarding antiplatelet, physicians can consider stopping P2Y12 inhibitors (clopidogrel, prasugrel, ticagrelor) at least 5 days prior to the surgery in patients considered to be at low risk for stent thrombosis, especially in patient with concomitant use of vitamin K antagonist or direct oral anticoagulant. Aspirin should be continued throughout procedure. In patients with high risk for in‐stent thrombosis, however, dual antiplatelet should be continued without interruption.^48^ Once hematoma develops, there is no evidence regarding the best approach to prevent CIED infection. From BRUISE CONTROL trial, it was unclear whether evacuation of hematoma was associated with any changes in risk of infection. More research and data are needed to answer this clinical problem.^13^

### 4.1 Limitation

Our meta‐analysis is not without limitations. First, despite the significant association we found on the main analysis, a causal relationship cannot be inferred. As mentioned earlier, certain patients may be at risk for both hematoma and CIED infection. Second, most data extracted from the included studies were not adjusted for confounders known to be associated with CIED infections, including diabetes mellitus, heart failure, renal dysfunction, oral anticoagulant, or long‐term corticosteroid use. In studies reported adjusted ratios, these factors were not uniformly addressed in addition to other factors such as definition of postimplantation hematoma, follow‐up duration, device type (pacemaker, defibrillator, biventricular defibrillator), and device revision/new implantation. There is also not enough sufficient data to perform subgroup analysis for these factors. Third, data from studies included in this meta‐analysis was obtained through a large time gap, from 1990 to 2017. There has been a major shift in practice, including protocol of the procedure and antithrombotic regimen in this population. Finally, our meta‐analysis reported combined CIED infections as the main result, which comprise local and systemic infection. Breaking down the outcome to local and systemic infections was not feasible because of the limited reported data from the original articles.

## 5 CONCLUSION

Our study suggested a statistically significant association between postimplantation hematoma and an increased risk of CIED infection following the implantation. This correlation should not be overlooked and extra steps to detect or prevent hematoma are needed to reduce CIED infection.

## CONFLICT OF INTEREST

All author declares no conflict of interests

## Supporting information

File S1

File S2
