# Supplementary material for: Postimplantation pocket hematoma increases risk of cardiac implantable electronic device infection: A meta‐analysis
Source: J Arrhythm. 2021 Mar 13;37(3):635–44. doi: 10.1002/joa3.12516 (PMC8207394; doi:10.1002/joa3.12516)
Supplement: Supplementary file 1 — File S1 [file JOA3-37-635-s002.docx]

Search term

PubMed

("haematoma"[All Fields] OR "hematoma"[MeSH Terms] OR "hematoma"[All Fields]) AND ("heart"[MeSH Terms] OR "heart"[All Fields] OR "cardiac"[All Fields]) AND implantable[All Fields] AND ("electronics"[MeSH Terms] OR "electronics"[All Fields] OR "electronic"[All Fields]) AND ("equipment and supplies"[MeSH Terms] OR ("equipment"[All Fields] AND "supplies"[All Fields]) OR "equipment and supplies"[All Fields] OR "device"[All Fields]) AND ("infections"[MeSH Terms] OR "infections"[All Fields] OR "infection"[All Fields])

EMBASE

'hematoma cardiac implantable electronic device infection' OR (('hematoma'/exp OR hematoma) AND cardiac AND implantable AND electronic AND ('device'/exp OR device) AND ('infection'/exp OR infection))
