# Supplementary material for: Postimplantation pocket hematoma increases risk of cardiac implantable electronic device infection: A meta‐analysis
Source: J Arrhythm. 2021 Mar 13;37(3):635–44. doi: 10.1002/joa3.12516 (PMC8207394; doi:10.1002/joa3.12516)
Supplement: Supplementary file 2 — File S2 [file JOA3-37-635-s001.docx]

| First author, year | Selection | | | | Comparability | Outcome | | | Total score |
| --- | --- | --- | --- | --- | --- | --- | --- | --- | --- |
|  | Representativeness | Selection of the non-exposed cohort | Ascertainment | Endpoint not present at start | Comparability | Assessment of outcome | Follow-up duration | Adequacy follow-up |  |
|  |  |  |  |  | (Confounding) |  |  |  |  |
| Ann, 2015 | * | * | * | * | * | * | * | * | 8 |
| Arana-Rueda, 2017 | * | * | * | * | * | * | * | * | 8 |
| Bloom, 2006 | * | * | * | * | * | * | * | * | 8 |
| Caldero, 2018 | * | * | * | * | ** | * | * | * | 9 |
| Cengiz, 2010 | * | * | * | * | ** | * | * | * | 9 |
| Essebag, 2015 | * | * | * | * | ** | * | * | * | 9 |
| Klug, 2007 | * | * | * | * | * | * | * | * | 8 |
| Korkerdsup, 2018 | * | * | * | * | ** | * | * | * | 9 |
| Olivia, 2009 | * | * | * | * | ** | * | * | * | 9 |
| Nery, 2010 | * | * | * | * | ** | * | * | * | 9 |
| Raad, 2012 | * | * | * | * | ** | * | * | * | 9 |
| Romeyer-Bouchard, 2009 | * | * | * | * | * | * | * | * | 9 |
| Sadeghi, 2018 | * | * | * | * | * | * | * | * | 8 |
| Uslan, 2012 | * | * | * | * | * | * | * | * | 8 |

Supplementary file 2: The Newcastle-Ottawa Scale of included studies

Notes: The Newcastle-Ottawa scale uses a star system (0 to 9) to evaluate included studies on 3 domains: selection, comparability, and outcomes. Star (*) = item presents. Maximum 1 star (*) for selection and outcome components and 2 stars (**) for comparability components. Higher scores represent higher study quality.
